# Friction and Wear of Tungsten Carbide Dies in the Dry Drawing of Steel Wire

**DOI:** 10.3390/ma18071409

**Published:** 2025-03-22

**Authors:** Maciej Suliga, Piotr Szota, Joanna Kulasa, Anna Brudny, Marek Burdek

**Affiliations:** 1 Faculty of Production Engineering and Materials Technology, Czestochowa University of Technology, Armii Krajowej 19, 42-201 Czestochowa, Poland; piotr.szota@pcz.pl; 2 Łukasiewicz Research Network—Institute of Non-Ferrous Metals, Sowińskiego 5, 44-100 Gliwice, Poland; joanna.kulasa@imn.lukasiewicz.gov.pl (J.K.); anna.brudny@imn.lukasiewicz.gov.pl (A.B.); 3 Łukasiewicz Research Network—Upper Silesian Institute of Technology, Miarki 12-14, 44-100 Gliwice, Poland; marek.burdek@git.lukasiewicz.gov.pl

**Keywords:** die, wire drawing, steel, friction, wear, die surface, fracture, FEM

## Abstract

This paper presents an analysis of the wear of the surface layer of drawing dies after the steel wire drawing process. It was shown that the working surface of the drawing die is characterized by high roughness combined with the occurrence of numerous scratches and blurs. As a result of high pressures in the deformation zone, premature wear of drawing dies combined with mechanical damage and the sticking of steel on the drawing surfaces can occur during the industrial drawing process. The finite element method analysis showed a significant relationship between the friction coefficient and the rate of drawing die wear. The varying distribution of stresses in the drawing die during the drawing process can contribute to mechanical damage. Longitudinal tensile stresses at the wire’s entrance to the drawing die increase the risk of circumferential cracking of drawing dies.

## 1. Introduction

Friction and lubrication affect drawing process conditions, tool life, and wire properties [1,2,3,4]. Depending on the technology adopted, the drawing process at the contact between the wire and the drawing die may involve dry friction (no lubricant) [5], semi-dry friction (a small amount of lubricant separating the friction surfaces) [6], semi-fluid friction (most of the load is transferred through the surfaces separated by grease and less directly through the friction surfaces) [7], and fluid friction (the wire and drawing die are separated by a layer of grease over the entire length of contact) [8,9,10]. At the same time, it can be assumed that dry, semi-dry, and semi-fluid friction conditions occur when drawing in conventional drawing dies, while fluid friction is obtained when drawing wires in hydrodynamic drawing dies, also described in the literature as pressure drawing [11,12,13]. These drawing dies consist of a retaining part, where the retracted lubricant particles melt due to friction, and a working part of the drawing die, where a melted layer of the lubricant under pressure separates the wire and the drawing die [14,15,16]. The most common application in industry is conventional drawing dies, in which mixed friction is introduced, i.e., in the zone of contact between the tool and the material; there are areas without grease, areas with a thin layer of grease, and areas where a thick film of grease is present. In the actual drawing process, both the drawing die and wire surfaces are not perfectly smooth and have different surface topographies; in such an arrangement, lubrication pockets are formed on the surface, where grease accumulates during the drawing process [17,18].

The lubricant used in dry multistage drawing is drawing powders with granular consistencies [19,20]. These lubricants differ in terms of chemical composition. For example, calcium lubricants are usually used in the first draws at low draw speeds (1–6 m/s), while in the other draws (at speeds above 6 m/s), sodium lubricants are used, characterized by greater thermal resistance [21]. An intermediate solution, which is often used in the practice of drawing, is the use of a mixture of calcium–sodium grease in all strings [22].

The use of the right combination of grease, drawing die, squish, and draw speed makes it possible to reduce the coefficient of friction by improving lubrication conditions [23]. In the literature, there are papers describing the influence of the shape of the working part of the drawing die on the energy and force parameters [24], lubrication conditions [25], surface topography [26], mechanical properties [27], and fatigue strength of wires [28]. The use of drawing dies with sigmoidal, curved, concave, and convex drawing die crush parts, compared to conventional conical drawing dies, results in improved lubrication conditions and lower drawing stress, and the wires drawn in these drawing dies exhibit higher properties [29]. Despite the unquestionable advantage of modified drawing dies over conical dies, these dies have not become popular in drawing plants. The main disadvantage of sigmoidal, concave, etc., drawing dies is the problem with maintaining the desired geometry. During the drawing process, as another ton of wire is pulled through, the drawing dies wear out, causing premature wear. Hence, the main direction in the development of drawing dies has been the optimization of the geometry of conical drawing dies, including the angle of drawing and the length of the calibrating part of the drawing die [30,31].

The literature shows that drawing technology affects the rate and mechanisms of tool wear in drawing processes [32,33,34]. A theoretical study conducted in [35] confirmed that in the drawing process, tool life depends on the drawing speed, temperature, drawing angle, and size of the squish. It has been shown that the wear rate of the drawing die results from several variables, which include the temperature of the wire and the drawing die, the effects of friction forces, and the unit pressure on the drawing die. As the drawing angle increases, the contact area between the material and the tool decreases, leading to a decrease in frictional forces and temperature on the one hand and an increase in the pressure of the wire on the drawing die on the other. When designing a multi-stage drawing process for the durability of drawing dies, it is necessary to adopt the principle that as the drawing speed increases in the successive draws, the size of the single squish and the angle of the working part of the drawing die decrease. Tribological studies in the sintered carbide/steel system included in the paper [35] confirmed that the average coefficient of friction increases with rising temperatures, and abrasion products are applied to both steel and tungsten carbide. Hence, it seems important to gain knowledge about the effect of the friction coefficient on the rate of wear of the drawing dies.

In this paper, the authors will focus their attention on analyzing the condition of the surface layer of drawing dies after the industrial steel wire drawing process. The examinations will be complemented with a theoretical analysis of the effect of friction conditions on the parameters of the drawing process and the wear of drawing dies.

## 2. Materials and Methods

### 2.1. Conical Drawing Dies with a Cemented Carbide Insert

The material for the study was conical drawing dies after an industrial wire drawing process with different degrees of wear. A total of 50 tons of wires were drawn in a drawing die with a diameter of dk = 1.7 mm. The average speed of wire drawing in the last draw was 15 m/s; the amount of wire drawn per hour was approximately 1 ton. The approximate working time of the tool is 50 h. In multi-stage drawing, the product of the drawing speed and the wire cross-section is constant. Therefore, in the case of a drawing die with a diameter of dk = 5 mm, the time to draw 5 tons of wire was 5 h.

Before drawing, the dies are polished, and their surface is characterized by high smoothness (profile deviation parameter Sa usually below 1 μm). After mounting the dies in the drawing box, filling it with grease, and then drawing a few meters of wire, the first scratches and abrasions appear on the surface of the dies.

These drawing dies were divided into two groups, i.e., dies showing typical abrasive wear of the working surface and dies with mechanical damage and geometric defects. Then, each of the drawing dies was cut into two parts using an electrosparking machine. In the first stage, a metallographic examination of the surface layer of the drawing dies was carried out using a Keyence VHX-7000 digital microscope (Keyence, Mechelen, Belgium). SEM studies were carried out on a Zeiss Gemini 1525 high-resolution scanning electron microscope (Zeiss, Aalen, Germany) equipped with a Quantax XFlash^®^ 6 Bruker Nano X-ray spectrometer (EDS SDD, Bruker, Billerica, MA, USA) along with EDS analysis of the chemical composition of selected areas of the tungsten carbide insert

### 2.2. Numerical Modeling of Steel Wire Drawing

Computer simulations of the wire drawing process were performed using the Simufact 2023.2 computer program and the finite element method. The research results presented in the previous work [35] showed that despite the cooling of the dies with water, after drawing about 10 m of wire, the temperature of the dies surface increases rapidly to over 500 °C. Hence, the initial temperature of the dies equal to 500 °C was assumed for the simulation of the stabilized wire drawing process. The experimental studies analyzed the mechanisms of the wear of dies with diameters of dk = 1.7 mm (the last draw) and dk = 5 mm (the first draw). The work showed that the dies with a diameter of dk = 5 mm, in addition to the typical wear for the drawing process, also showed mechanical damage in the form of a crack on its circumference. Hence, the work focused on the theoretical analysis of the wire drawing process from a diameter of 5.5 mm to a diameter of 4.75 mm (a single draft of 25% for the typical drawing process). The boundary conditions adopted for the simulation are as follows:

- material: C45 steel.
- wire drawing in a single sequence from a diameter of 5.5 mm to a diameter of 4.75 mm.
- conical drawing dies with the angle of 2 = 10°.
- drawing speed of 1 and 5 m/s.
- friction coefficients: 0.01, 0.04, 0.07, and 0.1.
- initial temperature: drawing die = 500 °C and wire = 50 °C.
- thermal conductivity of C45 steel: temperature-dependent.
- the dependence of the hardness of the cemented carbide on the temperature was taken from [36].
- carbide density: 14,700 kg/m^3^·s
- carbide thermal conductivity: 80 W/(m-K).
- carbide heat capacity: 0.2 J/(g-K).

Computer simulations made it possible to determine the effect of frictional conditions in the drawing process on the drawing stress, the wire and tool temperature, and the rate of wear of drawing dies.

## 3. Results

### 3.1. Tests of the Surface Layer of Drawing Dies

**Figure 1:**
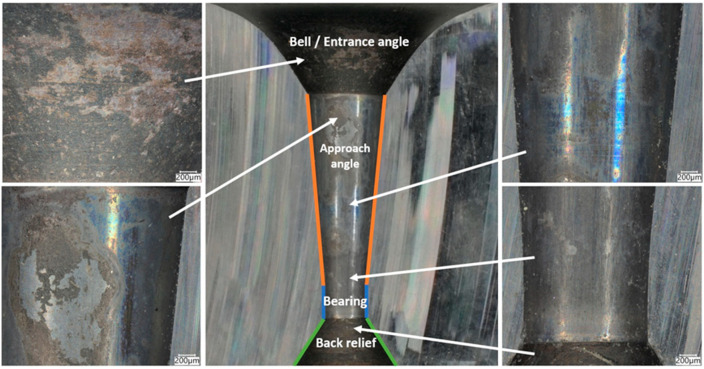
General view of the drawing die with a diameter of dk = 1.7 mm after drawing 50 tons of wire. Part I, typical wear after the drawing process.

**Figure 2:**
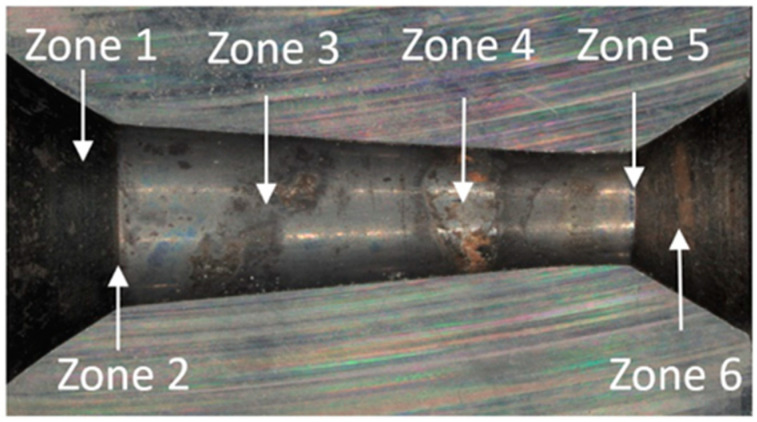
Drawing die with a diameter of dk = 1.7 mm. Part II with marked zones subjected to metallographic analysis.

Conical drawing dies in the drawing process must meet high-quality requirements with regard to dimensional tolerance, geometry, and surface smoothness. Hence, the technical inspection of tools consists of checking the convergence of cones, the parallelism of the calibration part, rounding at the transition of one part to the other, and material tests of the cemented carbide core (measurement of hardness, density, and surface topography). As another ton of wire is drawn, the surface of the drawing dies gradually wears down; scratches, scrapes, and, in special cases, cracks, among others, appear on their working surface. The first stage of this study analyzed a drawing die showing typical abrasive wear after the wire drawing process, as shown in Figure 1 and Figure 2.

**Figure 3:**
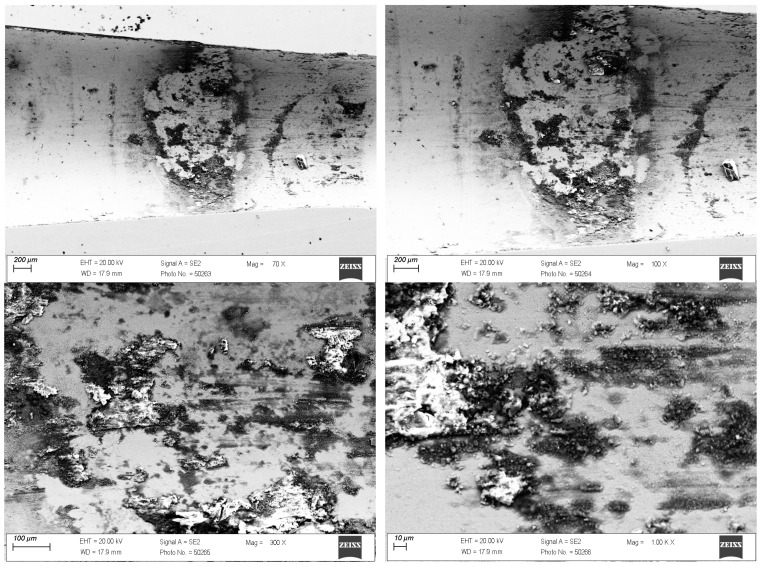
Drawing die surface with dk = 1.7 mm. Part II; zone 4.

**Figure 4:**
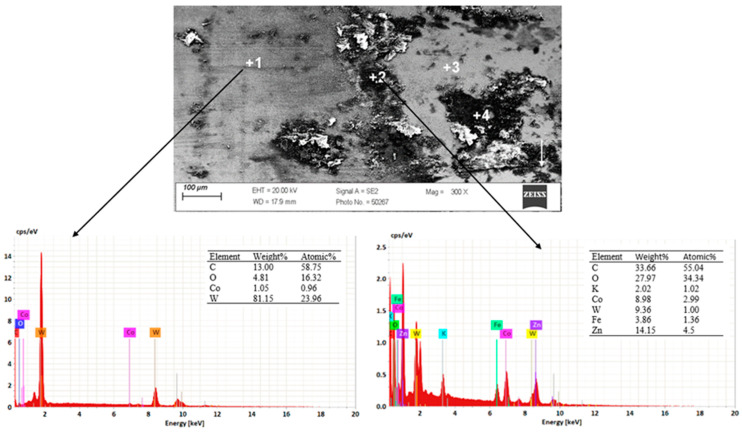
Results of EDS spot microanalysis of the composition in the area of the fracture with dk = 1.7 mm of the die; zone 3.

In the drawing process, there is a tribological node consisting of the wire, drawing die, and lubricant. Tool heating occurs as a result of plastic deformation of the wire in the deformation zone and friction at the wire/drawing die interface. The amount of heat generated during drawing depends on a number of factors, including the grade of the material being drawn, the geometry of the tools, the squish, and the drawing speed. As the amount of heat generated increases, the temperature of the drawing dies increases, causing accelerated wear. Hence, lubricants, usually calcium and sodium lubricants with a certain granularity, are used in order to lower the temperature of the drawing process. During drawing, hard grease particles are taken up by the wire into the drawing die. As a result of friction, they are gradually plasticized, and in the strain zone, they turn into liquid form. After exiting the drawing die, some of the grease is ejected outside, and the remainder is deposited and crystallized on the wire. The wire unwound from the drum is then directed to the next drawing box and drawing die, and during the transition, another new layer of lubricant is applied to the layer of the lubricant film coming from the previous sequence. In an ideal theoretical drawing process, a layer of lubricant evenly covers the friction surfaces of the tool and wire. In fact, in the process in question, there is an uneven distribution of lubricant thickness along the length of the strain basin, in which there are varying friction conditions. It leads to the formation of various mechanisms of drawing die wear. Hence, the tool wear process is complex and is related to abrasive wear, adhesion, thermal and mechanical fatigue, corrosion, and oxidation. By analyzing photos of the surfaces of typically worn drawing dies (Figure 1 and Figure 2), it can be seen that the degree and extent of the above-mentioned wear mechanisms vary depending on the analyzed part of the conical drawing die. The wire entry area of the drawing die (entry cone and lubricating cone) is mainly subject to wear related to corrosion, oxidation, and negligible abrasive wear, mainly from friction at the drawing die/grease interface. Hard particles of grease drawn by the wire into the entry zone of the drawing die as a result of friction become plasticized, and scratches and nicks gradually appear on the polished surface of the new drawing dies. In addition, there may be local contact between the wire and the drawing die in this area (the wire entering the drawing die has a variable curvature), which leads to slight abrasive wear. This is confirmed by the visible traces of corrosion, oxidation, and varying surface roughness seen in Figure 1 and Figure 2 (zone 2). The zone of the drawing die where all wear mechanisms occur is the crushing cone, referred to in the literature as the working part of the drawing die. In this area, there is an essential drawing process, i.e., strain of and reduction in wire diameter (zones 3 and 4). Hence, Figure 3 and Figure 4 show the drawing surface in zone 4 and EDS microanalysis of the chemical composition.

The conducted tests confirmed that the very high inhomogeneous wear of the crushing cone of the drawing die, in which material losses occurred as a result of abrasion, corrosion, and the oxidation of the surface, as well as the occurrence of micro-cracks and spalling, is prevalent. Figure 4 demonstrates that the condition of the surface layer, including surface roughness, can contribute to adhesive wear involving the formation of overgrowth (sticking). A microanalysis showed that the areas with smoother surfaces contain carbon, cobalt oxygen, and tungsten. In turn, in the area of greater surface degradation, additional iron, potassium, and zinc appeared. The increase in surface roughness creates additional resistance to the flow of metal in the drawing die, which leads not only to the deposition of abrasion products of carbide and steel but also to the detachment of microparticles from the wire surface and their direct deposition on the drawing die surface. The elements potassium and zinc are components of drawing grease, which tends to deposit on the surface of drawing dies.

**Figure 5:**
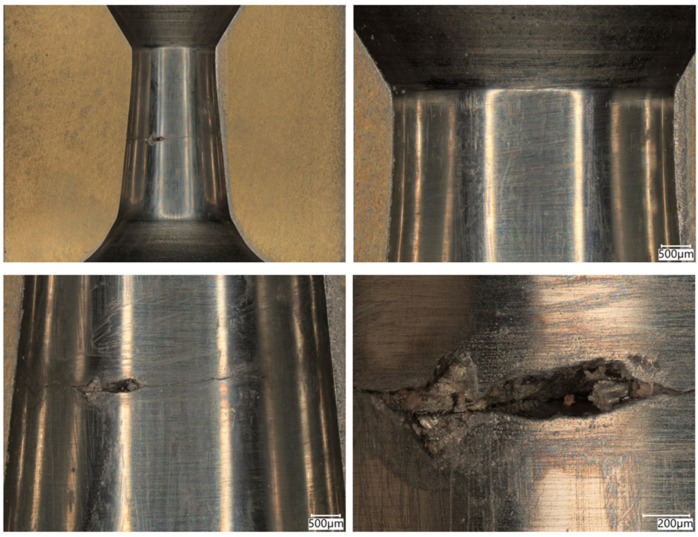
General view of the drawing die with a diameter of dk = 5 mm after pulling 5 tons of wire; mechanical failure of the drawing die.

The case discussed above is the typical wear of drawing dies after the process of drawing. In industrial practice, mechanical damage to drawing dies is also noted during multi-stage drawing, as shown in Figure 5.

**Figure 6:**
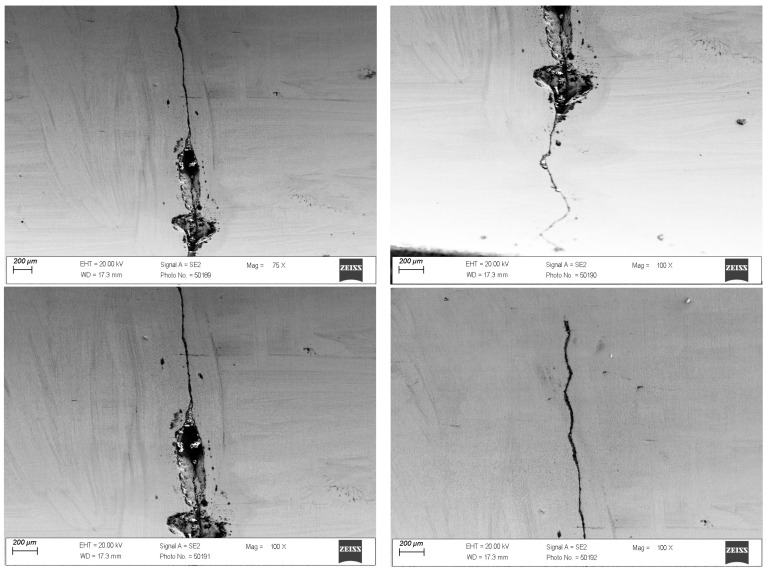
Surface of a drawing die with a diameter of dk = 5 mm in the fracture zone.

**Figure 7:**
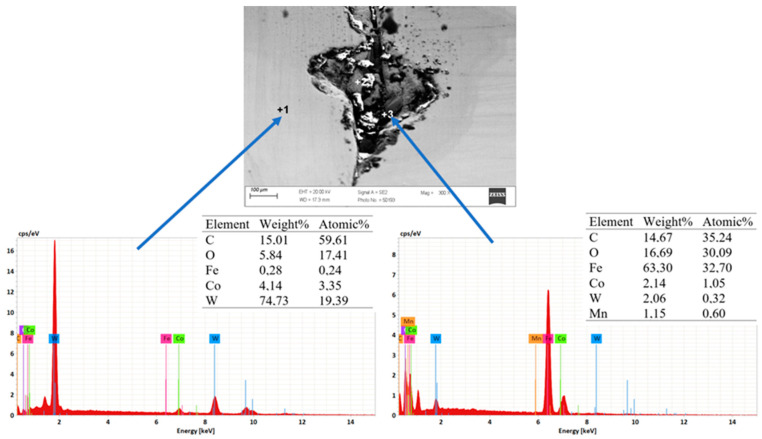
Results of EDS spot microanalysis of the composition in the area of the fracture with dk = 5 mm of drawing die loss.

The drawing die shown in Figure 5 is characterized by mechanical wear in addition to the typical wear shown in the previous figures. Metallographic observations of the drawing die in the input, cylindrical, and output zones showed initial signs of wear, i.e., scratches and nicks. In contrast, mechanical failure was the dominant wear mechanism in the crushing cone of the drawing die. In the drawing process, there is an alternating state of strain in the drawing dies as a result of the work of friction and plastic strain. The highest pressures occur at the wire–crushing cone interface (the beginning of wire strain) and at the transition of the wire from the crushing cone to the cylindrical part of the drawing die. The larger the diameter of the drawing die, the greater the forces acting on it. Accordingly, in order to protect the hard but brittle cemented carbide insert, steel housings are used when designing the drawing dies to reduce the risk of cracking the drawing dies’ cores. In most cases, the feedstock for drawing is a 5.5 mm diameter wire rod with a diameter deviation of ±0.2 mm. The case presented in Figure 5 refers to a drawing die with a diameter of dk = 5 mm, that is, a drawing die that is in the first stage of strain (sequence No. 1). In an ideal drawing process, the feedstock is round, while in reality, as a result of its ovality, a varying degree of strain and a singular squish at the perimeter of the wire are present. This leads to additional local stresses that increase the risk of cracking the cemented carbide core. According to the authors, this may have been the cause of cracking of the carbide insert on the periphery of the drawing die. Another cause of mechanical failure of the drawing die (Figure 5) may be a material and technological defect in the core created at the sinter production stage (an uneven distribution of the respective elements or poorly selected pressing and heat treatment parameters). Therefore, for a more complete analysis, an additional analysis of the drawing die from the fracture area was carried out. The results are presented in Figure 6 and Figure 7.

As in the case of the previously discussed drawing die with a diameter of dk = 1.7 mm, an EDS analysis of the chemical composition of the carbide in the fracture area showed the presence of elements such as oxygen, tungsten, cobalt, and iron. The presence of these elements confirms the presence of typical tool wear mechanisms. A small amount of iron, less than 0.3%, is the product of wire and carbide abrasion and the deposition of the abrasion product on the surface of the drawing die. In turn, the analysis of the fracture area with material loss showed the sticking of a large amount of iron in this area (locally, in the area with the largest crack, even more than 50% Fe). This can be explained as follows: With the wire passing through the drawing die, the crack in the macro carbide chipping was a barrier to the smooth flow of the steel in the drawing die. This resulted in an additional local increase in pressure and further propagation of the drawing die crack.

### 3.2. Computer Simulations of Wire Drawing

The study of the drawing dies after the drawing process, presented in the previous chapter of this paper, showed the presence of various wear mechanisms. The wear rate of drawing dies is affected by the parameters of the drawing process, including the coefficient of friction. Hence, the paper determines the effect of the friction coefficient on the temperature of the drawing die, surface pressures, drawing die wear, and the distribution of stresses in the drawing die.

**Figure 8:**
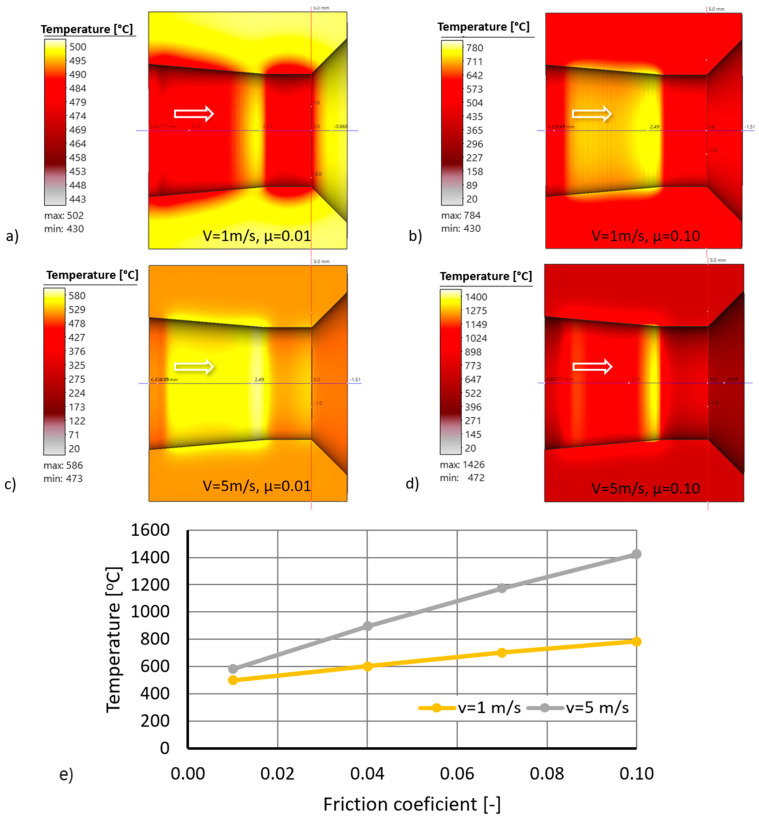
The effect of friction conditions on the surface temperature of drawing dies, when drawing wire from a diameter of 5.5 mm to a diameter of 4.75 mm, where (**a**) the drawing speed is 1 m/s and the friction coefficient is 0.01; (**b**) the drawing speed is 1 m/s and the friction coefficient is 0.1; (**c**) the drawing speed is 5 m/s and the friction coefficient is 0.01; and (**d**) the drawing speed is 5 m/s and the friction coefficient is 0.1. (**e**) The surface die temperature in the friction coefficient function.

**Figure 9:**
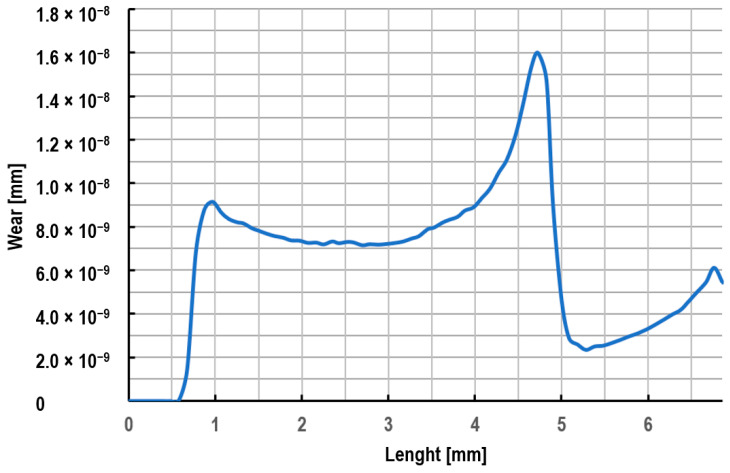
An example of the distribution of the wear of the drawing die along the length, when drawing the wire from a diameter of 5.5 mm to a diameter of 4.75 mm (a drawing speed of 5 m/s and a friction coefficient of 0.01).

By analyzing the data shown in Figure 8, it can be concluded that the coefficient of friction significantly affects the temperature on the surface of the drawing dies, and the higher the drawing speed, the greater the increase in temperature. Increasing the coefficient of friction from 0.01 (liquid friction corresponding to hydrodynamic drawing) to 0.1 (dry friction corresponding to wire drawing without a lubricant) at a drawing speed of 1 m/s resulted in an increase in the drawing temperature by 300 °C. It should be added that when drawing on multi-stage drawing machines, the wire drawing speed in the first sequence (e.g., from 5.5 mm diameter to 4.75 mm diameter) is usually 2–6 m/s. Numerical modeling shows that at a drawing speed of 5 m/s under dry friction conditions, the temperature of the drawing die at the wire/tool interface can rise to more than 1400 °C. Note that drawing the wire rod at such a high speed for dry friction conditions (m = 0.1) would generate a very significant heat flux due to plastic deformation and friction. As a result, in the actual drawing process, there would be thermal breakdown of the lubricant on the wire, the heating of the drawing surface (causing accelerated drawing wear and, in some cases, wire breakage), an increase in the drawing force, and wire breakage. The durability of a drawing die results from many factors, including temperature and unit pressure. These parameters change along the length of the contact between the wire and the drawing die; hence, the wear of the drawing die in the deformation basin is not a linear function, as shown in Figure 9.

**Figure 10:**
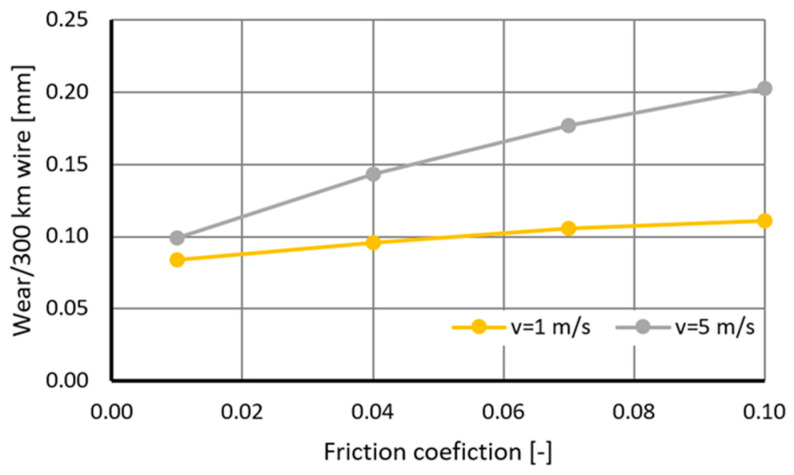
Influence of friction conditions on the drawing wear of drawing dies when drawing 300 km of wire from 5.5 mm diameter to 4.75 mm diameter, with the drawing speed of 5 m/s.

Figure 9 illustrates the distribution of the drawing die wear after pulling 0.03 m of wire. This distribution shows that the greatest wear occurs in the wire entry area of the drawing die and in the transition area from the crushing part to the calibrating part of the drawing die. In the area where the wire enters the drawing die, the factor determining the wear of the drawing die is the unit pressure, which is what contributes to the formation of the so-called crush rings in this area. In turn, the maximum wear of the drawing die occurs in the area of the transition from the crushing part to the calibrating part of the drawing die. This is caused not only by an increase in unit pressure but also by an increase in the temperature of the drawing dies and their decrease in abrasion resistance. Figure 10 shows the maximum wear of a drawing die after pulling 300 km of wire (about 37 tons of wire).

**Figure 11:**
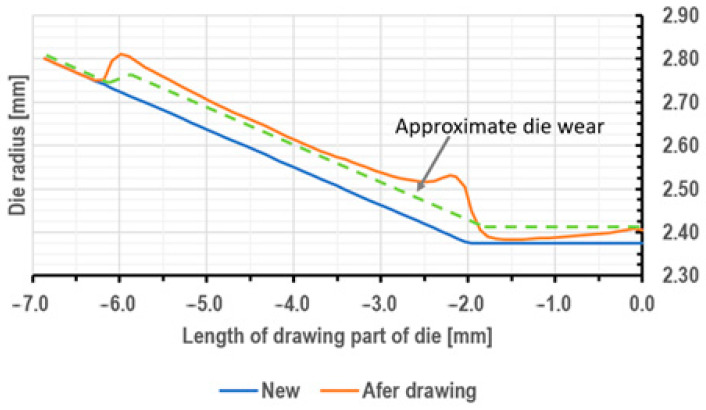
Wear distribution along the die length when drawing 300 km of wire from 5.5 mm diameter to 4.75 mm diameter, with the drawing speed of 5 m/s.

The data shown in Figure 10 shows that the coefficient of friction in the drawing process affects the wear rate of the drawing dies. A tenfold increase in the coefficient of friction from 0.01 to 0.1 resulted in an increase in drawing die wear, with draw speeds of 1 and 5 m/s by ca. 25% and 100%, respectively. At low drawing speeds, of the order of 1 m/s, the contact time between the wire and the drawing die is increased, and the heat generated by frictional forces is dissipated to a greater extent via conduction to the wire and less to the tool. Hence, there are smaller differences in drawing die wear. An increase in the drawing speed results in a greater amount of heat generated, a shorter time for the wire to pass through the drawing die, and an increase in heat accumulation in the surface layer of the drawing die, as shown in Figure 8. The amount of heat generated in the drawing process also depends on the diameter of the wire being drawn. The larger its diameter, the greater the work of plastic deformation and frictional forces. As a result, when drawing a wire with an initial diameter of 5.5 mm at a speed of 5 m/s, there was an approximately twofold increase in the maximum wear of the drawing die (in the area of transition from the crushing part to the calibrating part). The uneven distribution of drag wear is confirmed in Figure 11.

**Figure 12:**
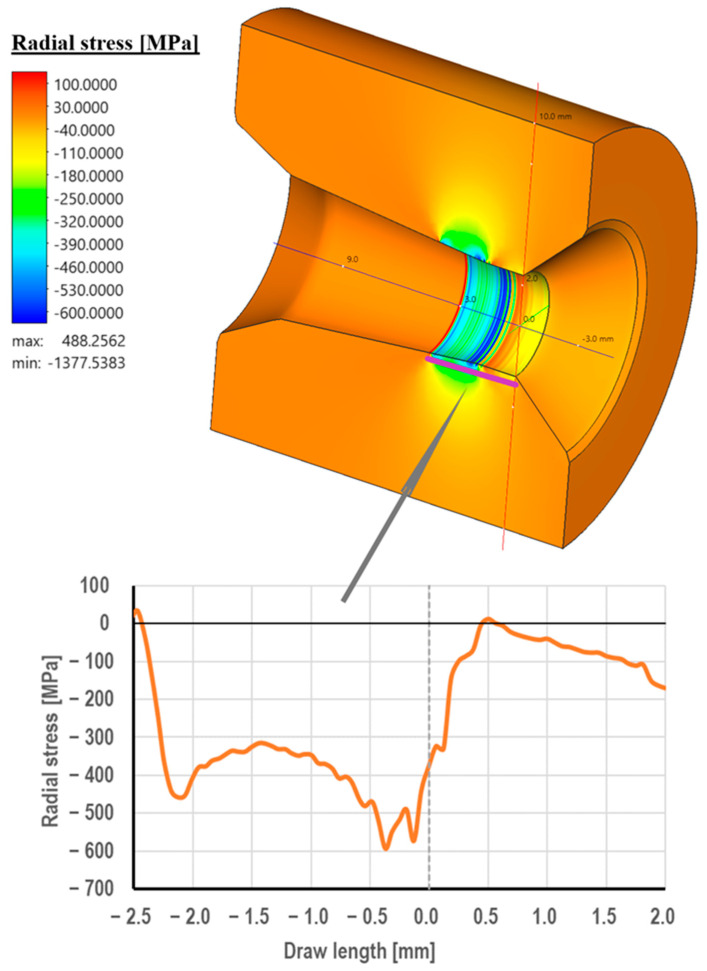
Distribution of radial stress in the drawing die when drawing wire from a diameter of 5.5 mm to a diameter of 4.75 mm (a friction coefficient of 0.07 and a drawing speed of 5 m/s)**.**

**Figure 13:**
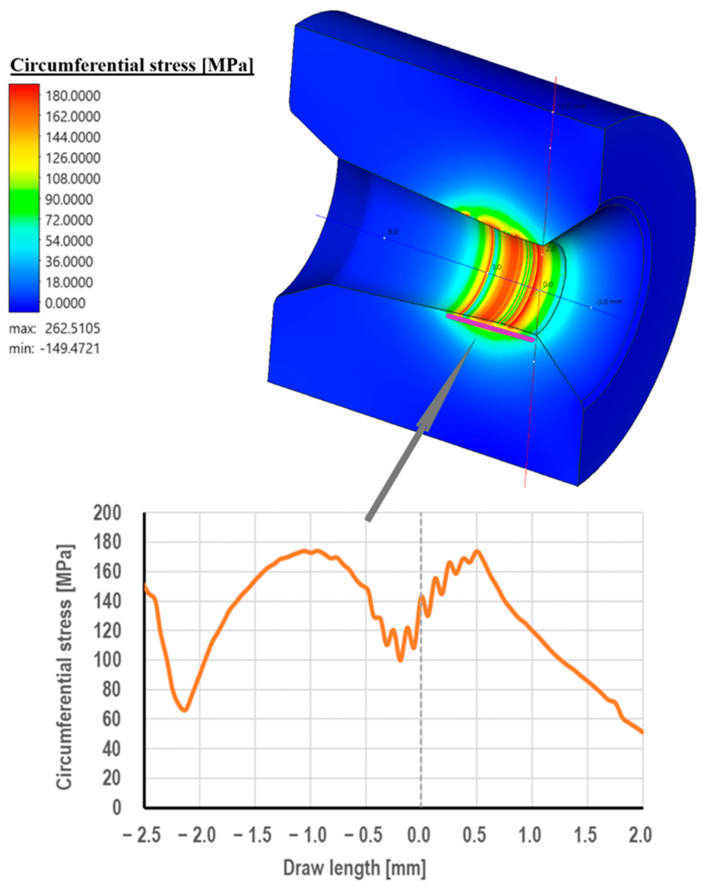
Distribution of circumferential stresses in the drawing die when drawing wire from a diameter of 5.5 mm to a diameter of 4.75 mm (a friction coefficient of 0.07 and a drawing speed of 5 m/s).

**Figure 14:**
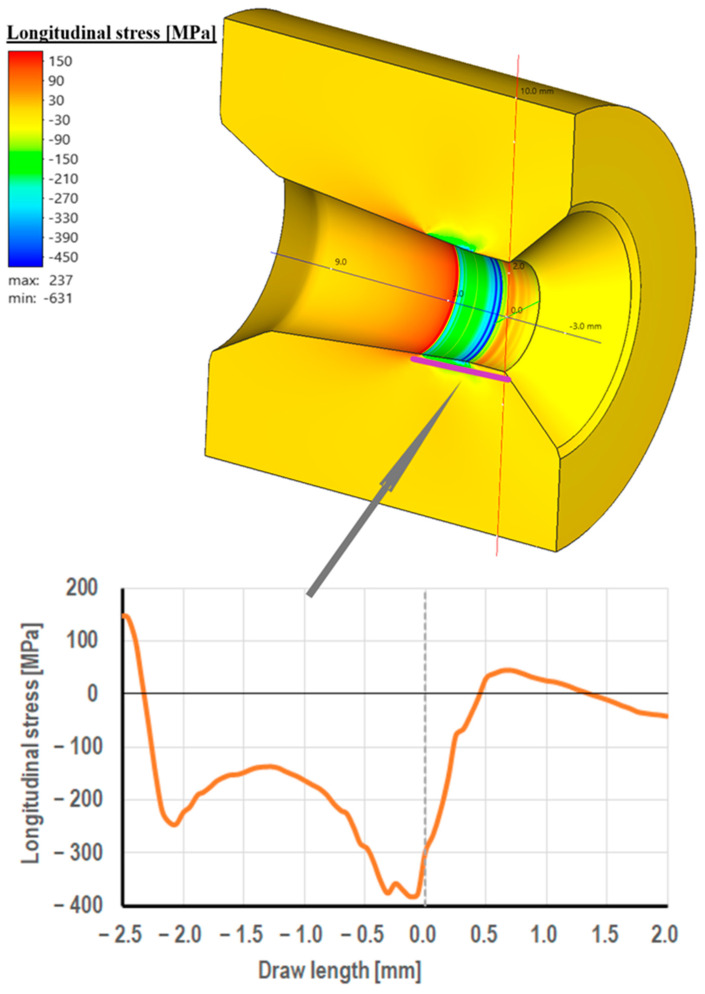
Distribution of longitudinal stresses in the drawing die when drawing wire from a diameter of 5.5 mm to a diameter of 4.75 mm (a friction coefficient of 0.07 and a drawing speed of 5 m/s)**.**

The mechanism of wear of drawing dies in the process of drawing is a complex issue, as uneven carbide abrasion in the deformation basin causes small local tool wear (the area of wire entry into the drawing die and the wire transition from the crushing part to the calibrating part). Subsequently, tool wear propagates in these areas along the direction of drawing. The simulation results presented in Figure 11 refer to theoretical tool wear after pulling about 37 tons of wire. In the actual drawing process, the curve depicting the worn drawing die has a different course (dashed line in Figure 11). In the area where the wire enters the drawing die, there are so-called crush rings that cause increased carbide abrasion. This leads to a concentration of stresses in this area of the drawing die, which can lead to cracking and wire breakage during the drawing process. Hence, the paper analyzed the distribution of stresses in the drawing die during the drawing process. The results of the calculations are shown in Figure 12, Figure 13 and Figure 14.

Drawing die cores are made of cemented carbide, usually in the K10 grade (94% W + 6% Co). This material is characterized by very high hardness and abrasion resistance. On the other hand, its biggest drawback is its brittleness. Hence, the drawing cores are placed in a steel frame for protection. Despite this, in the practice of industrial wire drawing, there are cases of mechanical damage to drawing dies, including their cracking. This is confirmed by the photographs of the drawing dies, as shown in Figure 5, Figure 6 and Figure 7. There are three stresses during the wire’s passage through the drawing die, i.e., radial stress, circumferential stress, and longitudinal stress. They are variable along the length of the deformation basin and can take on both positive and negative values. The distribution of radial stresses presented in Figure 13 showed that there are compressive stresses along approximately the entire length of the wire contact, with the highest values occurring in the area of the wire entrance to the calibrating part of the drawing die, ca. 600 MPa. In turn, local tensile stresses, not exceeding 50 MPa, can be observed in the area of the wire entrance to the drawing die.

Figure 13 shows that there are circumferential tensile stresses along the entire length of the drawing die. This can be explained as follows. As the wire is deformed in the drawing die, the forces acting on it try to “unravel” it and increase its circumference. Hence, tensile stresses occur on the surface of the drawing die in the material contact zone. The highest values were recorded in the middle part of the crushing cone and at the beginning of the calibrating part of the drawing die.

Regarding the longitudinal stresses along the direction of drawing, there are tensile stresses of about 150 MPa at the entrance to the drawing die. Subsequently, in the crushing cone of the drawing die, there is a change in the nature of longitudinal stresses, i.e., the disappearance of tensile stresses and the appearance of compressive stresses (−400 MPa, just at the entrance to the calibration part). Considering radial, circumferential, and longitudinal stresses, it can be concluded that in the area of the wire’s entrance to the drawing die, the dominant stresses are tensile. Thus, the circumferential fracture of the drawing die after the industrial drawing process shown in the paper (Figure 5) can be associated, among other things, with the occurrence of tensile stresses in the drawing die that expose the drawing mesh to fractures.

## 4. Conclusions

An analysis of the wear of drawing dies after the industrial wire drawing process showed uneven wear of the drawing die in the longitudinal direction and transverse to the drawing direction. The loss of material results from abrasion, corrosion, and the oxidation of the surface, as well as the occurrence of micro-cracks and chipping. In addition to the typical abrasive wear of drawing dies in the process of industrial wire drawing, mechanical damage to drawing dies, including circumferential cracking, can also occur. The analysis of the fracture area with the loss of the material showed the accumulation of a large amount of iron in this area (locally, in the area with the largest fracture, even more than 50% Fe). When the wire passes through the drawing die, a crack at the macro level caused by carbide chipping provides a barrier to the smooth flow of the steel in the drawing die. This results in an increase in additional local pressure and further propagation of the drawing die crack.

Computer simulations have shown that the coefficient of friction influences the life of the drawing dies. Increasing the coefficient of friction from 0.01 to 0.1 at a pulling speed of 5 m/s results in an increase in the wear of the drawing die by about 100%. The distribution of wear along the length of the drawing die is not a linear function. The highest values were recorded at the wire’s entrance to the drawing die and in the area of the wire’s transition into the calibrating part of the drawing die.

Considering radial, circumferential, and longitudinal stresses, it can be concluded that in the area of the wire’s entrance to the drawing die, the dominant stresses are tensile. Thus, the circumferential fracture of the drawing die after the industrial process of drawing can be associated, among other things, with the occurrence of tensile stresses in the drawing die that expose the drawing mesh to fractures.

## Data Availability

The original contributions presented in this study are included in the article. Further inquiries can be directed to the corresponding author(s).
